# Nyctalopia and Xerophthalmia in a Patient With Crohn’s Induced Vitamin A Deficiency

**DOI:** 10.7759/cureus.42961

**Published:** 2023-08-04

**Authors:** Zubair Khan, Victor Cox, Jack Creagmile, Aruoriwo Oboh-Weilke

**Affiliations:** 1 Department of Ophthalmology, Georgetown University School of Medicine, Washington, USA; 2 Department of Ophthalmology, Medstar Georgetown University Hospital, Washington, USA; 3 Department of Ophthalmology, MedStar Georgetown University Hospital, Washington, USA

**Keywords:** xerophthalmia, fat malabsorption, nyctalopia, crohn’s disease (cd), vitamin a deficiency

## Abstract

The vitamin A derivative, retinal, plays a pivotal role in scotopic and color vision. Although vitamin A deficiency (VAD) presents as a common cause of preventable blindness in areas with poor access to foods rich in vitamin A, it is uncommon in developed countries.

We present a 56-year-old male with a history of Crohn’s disease and pancreatitis who was referred to our ophthalmology office by optometry for severe dry eyes. He complained of a two-year history of constant blurred vision and nyctalopia. He stated that “images just appear dark.” Examination demonstrated mildly decreased visual acuity with severe ocular surface disease and characteristic Bitot’s spots in both eyes. Based on the patient’s history and physical, a diagnosis of xerophthalmia in the setting of VAD was made. The patient was referred to his internist, he then underwent further evaluation and treatment with vitamin A intramuscular injections post-diagnosis.

This case illustrates the potential for VAD secondary to malabsorption from Crohn’s disease and the importance of taking a full patient history so systemic causes of ophthalmic symptoms may be promptly identified and treated. VAD is extremely rare in the United States, however, patients at risk for VAD may benefit from regular vitamin A level checks and ophthalmologic evaluation.

## Introduction

Vitamin A is a lipid-soluble vitamin that is absorbed in the small bowel, primarily in the form of retinol. It is crucial to maintaining conjunctival and corneal epithelial integrity. It also plays a prominent role in the retina, allowing phototransduction in rod and cone cells. Vitamin A is found in dark leafy greens, milk products, liver, fish, as well as many other foods. It is absorbed in the duodenum after being hydrolyzed by pancreatic and intestinal enzymes and then emulsified by bile acids [1]. Therefore, a diet lacking in vitamin A or pathology that compromises the small intestine such as inflammatory bowel disease or bariatric surgery can result in a deficiency in vitamin A. Vitamin A plays a major role in vision, as it exists in the form of retinal to provide color and scotopic vision. Retinal serves as a requisite component of rhodopsin, the light-sensitive pigment found in the rod and cone cells of the retina, without which vision would be impaired. Moderate deficiency of rhodopsin as a result of vitamin A deficiency (VAD) leads to symptoms of nyctalopia while a severe deficiency results in xerophthalmia [2]. The most severe form of xerophthalmia is known as keratomalacia. In this ocular manifestation of VAD, spontaneous corneal melting via liquefactive necrosis occurs. This can cause rapid corneal degeneration and spontaneous corneal perforation [3].

The most common etiology of vitamin A deficiency worldwide is inadequate nutrition in resource-poor regions of the world complicated by chronic GI inflammation from recurrent infections. Zinc deficiency is another common cause of VAD worldwide, as it is required for the absorption of vitamin A and synthesis of retinol-binding protein (RBP), which facilitates the intravascular transport of retinol [4]. In areas in which it is endemic, measles has been shown to reduce serum retinol levels by greater than 30%. Measles accomplishes this by reducing the synthesis of RBP, which leads to increased secretion of vitamin A in the urine [5]. Other populations at risk of VAD worldwide are pregnant and lactating women residing in developing parts of the world due to increasing daily requirements of vitamin A in the setting of insufficient nutrient availability [6].

In contrast, VAD is extremely rare in the developed world due to an abundance of vitamin A-rich foods, greater sanitation, availability of clean water, and access to healthcare. VAD in the developed world is typically caused by various primary or secondary pathologies resulting in intestinal malabsorption. Inflammatory bowel disease (IBD), chronic liver disease, and pancreatic insufficiency are the common pathologies seen in cases of VAD in developed countries such as the United States. In the US, VAD is extraordinarily rare in the general population, estimated to be 0.3 % in 2013 [7]. Symptomatic VAD almost always involves a malabsorptive process or a severely restrictive diet. For example, 16% of children diagnosed with IBD in the U.S. have been diagnosed with VAD, with a higher prevalence in Crohn’s disease than in ulcerative colitis [8].

Seventy percent of patients with liver cirrhosis requiring transplant in the US experienced VAD. Furthermore, 35% of those experiencing chronic pancreatic insufficiency in the U.S. have been diagnosed with VAD despite treatment with pancreatic enzyme replacement therapy [9]. Bariatric surgeries have also been implicated in leading to VAD as they cause insufficient absorption of fat-soluble vitamins such as vitamin A. Premature neonates are at increased risk of VAD due to an immature GI tract, resulting in malabsorption of vitamin A.

An early eye-finding pathognomonic for VAD is Bitot’s spots, which are white/foamy lesions consisting of keratin. These lesions are typically seen on the bulbar conjunctiva adjacent to the limbus at the three o’clock and nine o’clock positions, with the temporal location being more common than the nasal [3]. The conjunctiva produces this keratin when VAD promotes squamous metaplasia due to the cells in the conjunctiva acting more like skin rather than a mucous membrane [3]. These spots are usually reversible if detected early but can lead to irreversible visual deficits if persistent. Long-standing VAD that has led to extensive corneal scarring from ulceration or keratomalacia also usually results in an irreversible visual disturbance [7].

While uncommon in developed countries, VAD presents as a leading cause of preventable reversible and irreversible blindness in areas of poverty, limited development, and poor availability of foods rich in vitamin A [10]. Vitamin A deficiency can also lead to an increased risk of infectious diseases through the impairment of innate immunity [10]. The aforementioned complications of VAD can result in increased mortality risk and contribute significantly to the global burden of VAD.

## Case presentation

**Figure 1:**
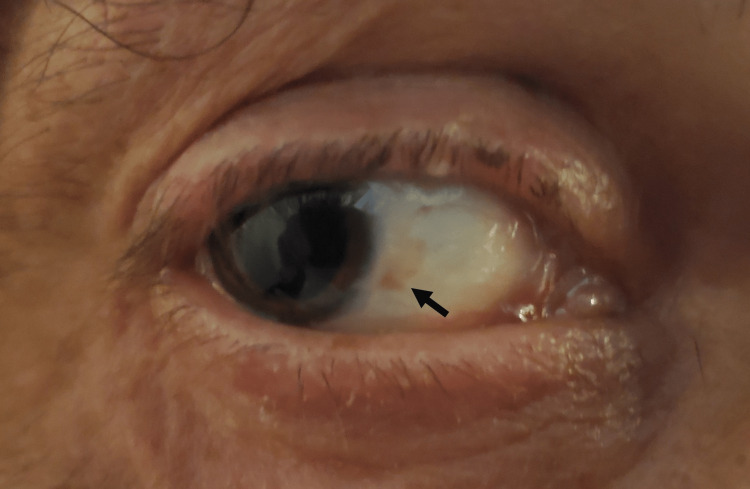
Unstained picture of Bitot’s spot in the patient's right eye

**Figure 2:**
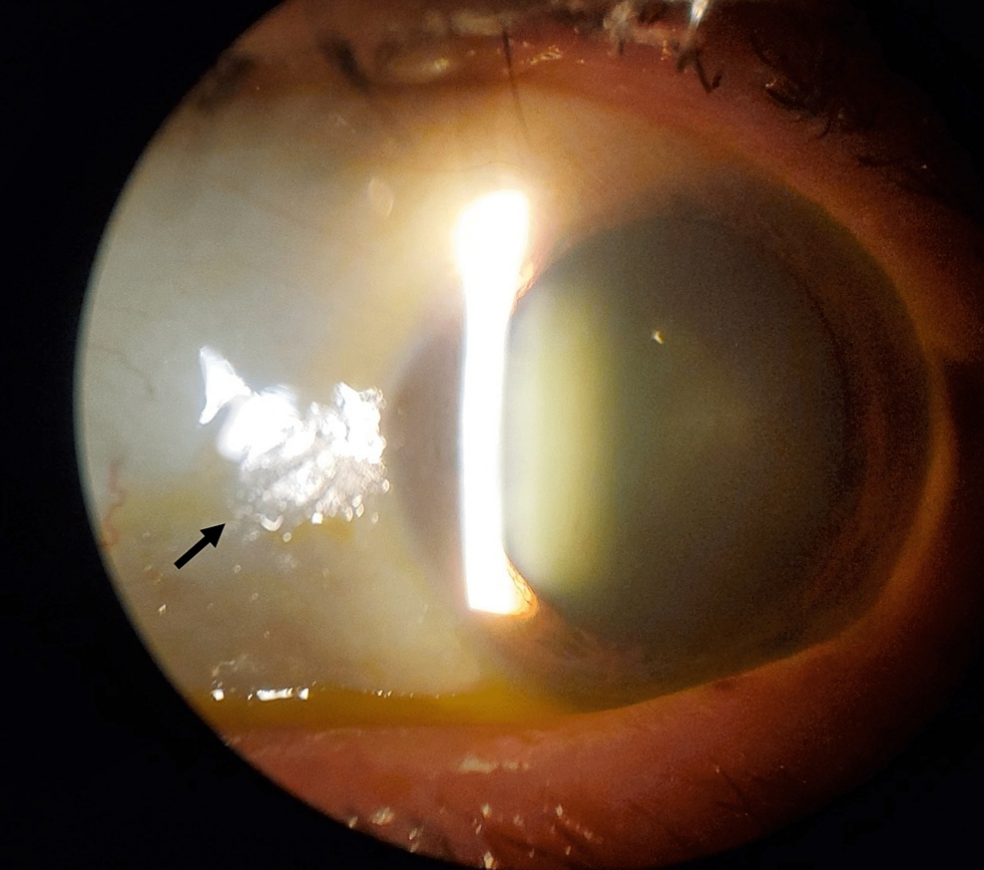
Slit lamp examination of the right eye showing Bitot’s spots in the temporal limbus with some glistening

We present a 56-year-old male with a history of Crohn’s disease and pancreatitis who was referred to ophthalmology by an outside optometrist for severe dry eyes, two years of bilateral blurred vision, and progressive night blindness. He was not on maintenance therapy for Crohn’s at the time. He complained of constant blurred vision in both eyes and stated that “images just appeared dark” and that he had trouble seeing when going from a light room to a dark room. He previously used lubricants as directed, hourly in both eyes. On examination, the patient had a visual acuity of 20/25 -1 right eye and 20/20 left eye, with equal, round, and reactive pupils. Intraocular pressures were 16 in the right eye and 13 in the left, with full extraocular movements. Slit lamp examination revealed triangular, glistening Bitot’s spots on the nasal and temporal limbi in both eyes, with bilateral punctate epithelial erosions (PEE) 4+ in the right eye and 3+ in the left, based on the ocular surface staining grading system (Figures 1, 2). The remainder of the anterior segment and fundus examination were unremarkable and non-contributory. Based on the patient’s history and clinical findings, a diagnosis of xerophthalmia in the setting of vitamin A deficiency was made. The patient was strongly encouraged to follow up with his primary care physician and referred to a retina specialist for further evaluation. He underwent treatment with vitamin A intramuscular injections post-diagnosis with his primary care physician. The patient did not continue follow-up.

## Discussion

This case report serves to describe a rare case in the U.S. of nyctalopia and xerophthalmia in the setting of vitamin A deficiency in a patient with chronic malabsorption due to Crohn’s disease.

The World Health Organization has recommended the administration of 200,000 IU of vitamin A orally, followed by a repeat dose the following day and another dose two weeks later to manage VAD. In cases of chronic malabsorption, such as our patient’s, WHO recommends administering vitamin A intramuscularly rather than orally [11]. Improvement of Bitot’s spots is expected to occur within two weeks and frequent follow-up is recommended to ensure resolution of symptoms [12]. However, night blindness and poor dark adaptation often persist for at least four weeks due to delayed response to treatment in the retina. In addition, it is recommended that the patient be treated with preservative-free artificial tears as well as antibiotics if a secondary bacterial infection exists. Any patient with active xerophthalmia warrants a thorough check for infectious disease due to their immunodeficient state. Nonetheless, treatment with vitamin A can restore their immune system and help prevent future infections [13].

Management of dietary vitamin A deficiency relies on proper nutritional supplementation, rehabilitation, and as well as education. Efforts should be made to provide education on vitamin A-rich foods such as eggs, beef, chicken, yams, whole milk, and carrots [11]. To address VAD caused by non-dietary causes, it is important to address underlying diseases such as liver disease as well as inflammatory bowel disease. As low serum levels of vitamin A do not necessarily indicate a clinically significant deficiency, educating patients on the ocular manifestations of vitamin deficiency is important. For asymptomatic patients, working toward controlling the patient's underlying Crohn’s disease and ensuring the patient receives the recommended daily intake of vitamin A should be prioritized [14]. The importance of controlling the underlying disease is demonstrated by this case, as the patient was not receiving systemic maintenance therapy at the time of presentation.

This case illustrates the potential for the development of ocular manifestations of VAD secondary to malabsorption from Crohn’s disease. This risk highlights the importance of taking a full patient history so that systemic disease can be promptly identified and treated.

## Conclusions

Although VAD is quite rare overall in the United States, there are various subgroups of patient populations that are at relatively high risk of developing VAD. In order to prevent the development of potentially severe and vision-threatening ocular manifestations of VAD, it is essential that these high-risk patients are identified and the underlying disease is managed.
